# Ipilimumab treatment enhances CD4+ T cell activation while decreasing Treg and MDSC frequency in advanced melanoma patients

**DOI:** 10.1186/2051-1426-2-S3-P231

**Published:** 2014-11-06

**Authors:** Yago Pico de Coaña, Isabel Poschke, Yuya Yoshimoto, Sara Karlberg, Maria Nyström, Maria Wolodarski, Johan Hansson, Giuseppe Masucci, Rolf Kiessling

**Affiliations:** 1 Karolinska Institute, Stockholm, Sweden; 2 German Cancer Research Center, Germany

## 

Ipilimumab is a fully human antibody that blocks CTLA-4 and has proven to extend overall survival in patients with unresectable stage III or stage IV melanoma. There is a need for well-documented pharmacodynamic markers together with potential predictive biomarkers that may allow for pretreatment selection of patients and screening for IRAE. Most of the recently published immune monitoring studies focus mainly on the effect that Ipilimumab has on T cell populations. To date, little information is available on the possible impact that Ipilimumab treatment may have on MDSC populations and their suppressive mechanisms. In order to evaluate these effects, we conducted an in-depth immune monitoring study centered on peripheral blood MDSC populations as well as T cells in advanced melanoma patients undergoing treatment with Ipilimumab.

Six patients with advanced stage melanoma received Ipilimumab treatment at 3 or 10 mg/kg doses as part of an ongoing double blind randomized trial (BMS trial CA184-169). Twenty-two additional patients received 3 mg/kg doses. Blood samples were collected from each patient before treatment and at the time of the second and fourth Ipilimumab doses.

ICOS^+^CD4^+ ^T cell frequency was significantly increased after Ipilimumab treatment, suggesting an increase in the activation of this cellular population. Analysis of circulating Treg frequencies revealed an initial increase, significantly decreasing after the second Ipilimumab dose. The endpoint mean frequency of Tregs was significantly lower than the baseline.

Changes in MDSC populations with granulocytic and monocytic phenotype (Lin^-^HLA^-^DR^-/lo^CD15^+ ^CD33^+^CD11b^+ ^and Lin^-^HLA^-^DR^-/lo^CD14^+^, respectively) were monitored and the results showed that after the first dose, the granulocytic MDSC population significantly decreased, remaining low at week 9. This decrease was accompanied by a significant decrease in the population of ARG1^+ ^myeloid cells.

Further analysis showed that the populations of granulocytic MDSCs and CD4^+^ICOS^+ ^T cell populations presented a statistically significant correlation.

These results provide a first look at the early responses of peripheral blood myeloid cell populations to Ipilimumab treatment. CTLA-4 blockade may be acting upon the granulocytic MDSC population reducing its frequency and functionality as soon as three weeks after administration of the first Ipilimumab dose. The mechanisms by which these *in trans *effects are taking place should be further explored as well as their possible relations to clinical benefit.

## Consent

Written informed consent was obtained from the patient for publication of this abstract and any accompanying images. A copy of the written consent is available for review by the Editor of this journal.

